# The bidirectional interation between pancreatic cancer and diabetes

**DOI:** 10.1186/1477-7819-10-171

**Published:** 2012-08-24

**Authors:** Junhui Li, Gang Cao, Qingyong Ma, Han Liu, Wei Li, Liang Han

**Affiliations:** 1 Department of General Surgery, Second Affiliated Hospital, School of Medicine, Xi’an Jiaotong University, 157 West 5th Road, Xi’an, 710004, People’s Republic of China; 2 Department of Hepatobiliary Surgery, First Affiliated Hospital, Medical College, Xi’an Jiaotong University, 277 Yanta West Road, Xi’an, 710061, People’s Republic of China

**Keywords:** Review, Pancreatic cancer, Diabetes, Bidirectional interation

## Abstract

The bidirectional interation between pancreatic cancer (PanCa) and diabetes has been confirmed by epidemiological studies, which provide evidence-based medical support for further research into the mechanisms involved in the interaction. We reviewed the literature regarding the role of diabetes in the generation and progression of PanCa and the mechanism by which PanCa induces diabetes for its malignant progression. The effect of antidiabetic drugs on the occurrence and prognosis of PanCa was also reviewed. Diabetes may directly promote the progression of PanCa by pancreatic duct enlargement and hypertension, as well as by enabling an increased tumor volume. Hyperinsulinemia, insulin resistance, cytokines, hyperglycemia and genotype change are also important factors in the progression of PanCa with diabetes. Hyperglycemia may be the first clinical manifestation and is helpful in the early diagnosis of PanCa. Furthermore, antidiabetic drugs can have different effects on the occurrence and prognosis of PanCa. The bidirectional interation between PanCa and diabetes is involved in the occurrence, proliferation, invasion, metastasis and prognosis of PanCa with diabetes. The discovery of biomarkers for the early diagnosis of PanCa, as well as the novel usage of metformin for its antitumor effects and determining the potential mechanisms of these effects, may be the next direction for PanCa research and treatment.

## Review

### Introduction

Pancreatic cancer (PanCa) ranks fourth in the United States and eighth to ninth worldwide among cancer-related deaths. From 1999 to 2008, the incidence rate of PanCa increased among men and women. During the same period, death rates increased for pancreatic cancer among both men and women. PanCa is more common in elderly persons aged 60 years and older, with fewer than 7% of patients presenting with localized, potentially curable tumors and approximately 53% of patients presenting with distant metastases. The overall five-year survival rate among patients with pancreatic cancer is <6% [1,2].

The bilateral causality between PanCa and diabetes has been widely documented. Type 2 diabetes could be either a cause, and/or possibly a consequence of PanCa [3].

### Increased incidence of PanCa in diabetic patients

Epidemiological studies have confirmed a higher incidence of PanCa in diabetic patients. A meta-analysis of cohort studies reported that individuals in whom diabetes had only recently been diagnosed (<1 year) had greater risk of malignancy in both males and females and that diabetes mellitus (DM) is both an early manifestation and an etiologic factor of pancreatic cancer [4]. Li *et al*. explored the association between diabetes and risk of PanCa in 2,192 cases and 5,113 controls in three large case–control studies using the method of multivariable analyses and found that diabetes was associated with a 1.8-fold risk of PanCa [5]. In a cohort study, Ogunleye *et al*. found the risk ratio to be significantly increased for pancreatic, liver and colon cancer [6]. Magruder *et al*. also reported that long-standing diabetes increases the risk of pancreatic cancer by 40% to 100%. Recent-onset diabetes is associated with a 4- to 7-fold increase in risk, such that 1% to 2% of patients with recent-onset diabetes will develop pancreatic cancer within three years [7]. Johnson *et al*. performed a population-based retrospective cohort study and found that within three months following diabetes onset, diabetics had a significantly increased risk of PanCa [8]. A population-based cohort study of 37,926 child-bearing Israeli women with a 28- to 40-year follow-up for diabetes and PanCa showed a 7-fold increase in the relative risk for PanCa development in women with a history of gestational diabetes mellitus. Furthermore, glucose levels in the upper range of normal have been associated with an increased risk for the development of some cancers, including PanCa [9]. Several studies have confirmed type II diabetes as a risk factor for PanCa. But rare research about the relationship between dyslipidemia and PanCa is reported. Tseng presented a new viewpoint that dyslipidemia was a significant risk factor for PanCa with a random sample of 1 million subjects, which would be helpful for clarifying the mechanisms of metabolic syndrome on PanCa [10].

A regional disparity was also found in diabetes-associated PanCa. For example, Hispanic men and Asian individuals had a higher risk of diabetes-associated PanCa than did white and black individuals, although the differences were not statistically significant [5]. Ogawa *et al*. found that in Japan the prevalence of PanCa in a group of individuals diagnosed with recent-onset diabetes (within three years) was 13.6%, which was significantly greater compared with the group of individuals who were diagnosed with diabetes more than three years prior (2.0%) [11]. Hong *et al*. reported a prevalence of PanCa among diabetic patients of 1.6% and a prevalence of diabetes in PanCa patients of 40.6% in Korea [12]. Chari *et al*. reported that approximately 1% of diabetics aged ≥50 years will be diagnosed with pancreatic cancer within three years of first meeting the criteria for diabetes. Furthermore, in 2,122 individuals in the USA, 10 of 18 (56%) were diagnosed <6 months after first meeting the criteria for diabetes [13]. An additional study in Americans analyzed a total of 1,172,496 case and control subjects and found that the frequency of pancreatic cancer in subjects with diabetes was 0.9% in comparison to subjects without diabetes (0.3%) [14].

Stevens *et al*. conducted a systematic review of the risk of pancreatic cancer in people with type 1 and young-onset diabetes, and found an increased risk of pancreatic cancer in type 1 diabetics, based on 39 cases of pancreatic cancer in young-onset and type 1 diabetes [15].

### Increased incidence of diabetes in PanCa patients

Tumor removal in PanCa patients improved glucose metabolism, which may suggest the presence of diabetogenic effect of PanCa [16]. Diabetes, especially new onset, is more frequent in PanCa patients and has been considered an early manifestation for asymptomatic PanCa. Approximately 80% of PanCa patients have glucose intolerance or frank diabetes [17]. In a population-based case–control study of PanCa, participants with PanCa were more likely to report a history of diabetes (13%) and had a shorter duration of diabetes and a larger proportion of insulin users than the control groups [18]. Diabetes has a high (50%) prevalence in PanCa and is frequently new onset (<36 months before index) in subjects with PanCa compared with subjects without PanCa [19]. At the time of diagnosis, 80% of PanCa patients have either impaired glucose tolerance or diabetes [20]. The incidence of diabetes is 34.63% among PanCa patients in China, of which 74.56% experience diabetes onset within two years of cancer diagnosis [21]. Matsubayashi *et al*. found the patients with familial PanCa were more likely to have diabetes mellitus (32.5%) than sporadic cases [22]. However, Bartsch *et al*. reported there were no statistical significant differences between familial and sporadic PanCa cases regarding the presence of diabetes and that diabetes was not a risk factor for pancreatic cancer [23,24].

### Bilateral mechanism between PanCa and diabetes

There has been a paradigm shift in the approach to understanding the relationship between PanCa and diabetes, as research has progressed into identifying the potential mechanisms that diabetes drive PanCa (Table 1).

**Table 1:** The potential mechanisms of diabetes in PanCa

**Parameters**		**Role in PanCa**	**Refs**
Pancreatic duct	Enlargement	Mechanical obstruction	[24]
	Replication increased	Predisposing factor	[25]
Hyperinsulinism and insulin resistance	Post-insulin receptor defect	Impaired glycogen synthesis and storage	[27]
	Plasma glucagon levels and IAPP	Early development of PanCa	[28]
	Polymorphism of -23HphI (A/T)	Pathogenesis of PanCa	[29]
Tumor size	Increased	Decreased postresection survival	[31]
		Enhanced growth	[32]
Genetic variants	HK2 R844K GA/AA genotype	Increased risk	[33]
	K-ras codon 12 mutations	Increased risk	[34]

*IAPP*, islet amyloid polypeptide; *PanCa*, pancreatic cancer.

#### Pancreatic duct enlargement and hypertension

Pancreatic duct enlargement and hypertension are more common in PanCa patients with diabetes than those without. Furthermore, mechanical obstruction of the pancreatic duct may be involved in the pathophysiology of diabetes in patients with PanCa [25]. Of note, using a high-fat diet-fed rat model of type 2 diabetes, Butler *et al*. noted that exocrine duct replication is increased tenfold by obesity and fourfold by diabetes, which is a predisposing factor for pancreatitis and PanCa [26].

#### Hyperinsulinemia and insulin resistance

Diabetes is usually characterized by profound peripheral insulin resistance in PanCa patients. Schneider *et al*. found that although half of the hamsters in their high-fat group developed malignant lesions with increased hyperplasia, the premalignant lesions were found within the islets. This finding may explain why the association between PanCa and obesity is usually associated with peripheral insulin resistance [27].

The intracellular mechanism of insulin resistance in PanCa has been investigated. Liu *et al*. found multiple defects in glycogen synthesis in PanCa patients with or without diabetes. Furthermore, the fractional velocity of glycogen synthase was decreased, whereas glycogen phosphorylase a and b activities were increased, in diabetic PanCa patients. However, glycogen phosphorylase mRNA levels were not significantly different, meaning that the insulin resistance associated with PanCa is associated with a post-insulin receptor defect, which impairs skeletal muscle glycogen synthesis and glycogen storage, leading to the hyperglycemia commonly observed in relation to PanCa [28]. While investigating islet function and secretion during early development of PanCa, Permert *et al*. found that plasma glucagon and islet amyloid polypeptide (IAPP) were significantly increased at 12 and 27 weeks, respectively, showing that islet hormone changes accompany the early development of pancreatic tumors in the hamster pancreatic cancer model [29]. Krechler *et al*. have studied the associations of the single nucleotide polymorphism -23HphI, which neighbors the variable number of tandem repeats locus in the upstream promoter of the insulin gene (INS), with PanCa and type 2 diabetes, and found that polymorphism of -23HphI (A/T) may play a role in the pathogenesis of PanCa and could contribute to tumor staging [30].

#### Tumor size increased by diabetes

Tumor size is an important prognostic factor for PanCa, as patients with small tumors have better prognosis and survival after surgery than those with large tumors [31]. Chu *et al*. examined the records of resected PanCa patients and found that preexisting diabetes is associated with reduced survival in patients undergoing resection for PanCa. Additionally, PanCa with new-onset diabetes may exhibit increased tumor size and decreased postresection survival [32]. When hamster H2T pancreatic carcinoma cells were implanted into the cheek pouches of Syrian hamsters, tumor size, tumor weight, and total tumor DNA content were significantly greater in animals with diabetes, which shows that diabetes appears to promote the growth of PanCa cells in the hamster [33].

#### Genotype and the progression of PanCa in diabetes

Dong *et al*. hypothesized that genetic variants in glucose metabolism modify individual susceptibility to PanCa, especially those associated with diabetes, and retrospectively genotyped 26 single-nucleotide polymorphisms in five glucose metabolism genes. The *HK2* R844K GA/AA genotype was associated with a reduced risk of PanCa among nondiabetic individuals but with increased risk among diabetic patients. Their findings show a potential role of the *HK2* gene, alone or in combination with diabetes, in modifying the risk of PanCa [34]. In a case–control study, Fryzek *et al*. found that diabetes diagnosed five or more years prior was associated with pancreatic cancer that was positive for K-ras codon 12 mutations, but not meaningfully related to patients with p53 mutations, though further large-scale studies are warranted [35].

Subsequent studies have identified a multitude of molecules, for which expression was altered in cancer cells, thus suggesting a potential role for these molecules in PanCa (Table 2).

**Table 2:** Molecular factors involved in progression in PanCa cells related to diabetes

**Molecules**	**Expression levels**	**Role in PanCa**	**Refs**
IGF-1 and receptor	Increased	Increase the risk and worse survival	[35,36]
14-amino-acid peptide from S100A8	Increased	Promote the growth of PanCa by activating Akt and NF-κB signaling	[38,39]
Regenerating gene I alpha protein	Increased	Accelerated cell proliferation	[40]
	Increased	Predictors of poor survival	[41]
	Increased	Contribute to determining diabetes	[42]

*IGF-1*, insulin-like growth factor 1; *PanCa*, pancreatic cancer.

Insulin-like growth factor 1 (IGF-1) and IGF-1 receptor are highly expressed in pancreatic cancer cells [36]. Suzuki *et al*. showed that polymorphic variants of the IGF genes alone or in concert with diabetes increase the risk of PanCa [37]. Thus, individual genetic variations in the IGF axis may predict worse survival in patients with PanCa [38]. Basso *et al*. isolated a 14-amino-acid peptide from S100A8 in PanCa tissue from diabetics and found that NT-S100A8 exerts a mild effect on PanCa cell growth in BxPC3, while it reduces PanCa cell invasion in MiaPaCa2 and Panc1, possibly by activating Akt and NF-κB signaling [39,40]. Furthermore, Zhou *et al*. found that the regenerating gene (REG) I-alpha protein was preferentially expressed in cancerous tissues and cancer cells of PanCa patients with diabetes and that overexpression of this protein resulted in accelerated cell proliferation and consequently tumor growth, both *in vitro* and *in vivo*[41]. A systemic inflammatory response is often observed in PanCa. For example, C-reactive protein was an independent predictor of survival [42]. Furthermore, circulating levels of several cytokines were high in patients with pancreatic carcinoma. Interleukin 6, which is released in large amounts by the inflamed pancreas in PanCa, may contribute to diabetes [43].

The association between diabetes and PanCa has long been recognized as that long-standing diabetes is thought to be an etiologic factor for PanCa and new-onset diabetes mellitus may be a manifestation of the cancer, both of which are characterized by hyperglycemia. The potential mechanisms of hyperglycemia in PanCa are shown in Table 3.

**Table 3:** The potential mechanisms of hyperglycemia in PanCa progression

**Parameters**		**Expression levels**	**Role in PanCa**	**Refs**
RAGE		Increased	Increased metastatic ability	[44,45]
ROS	Hydrogen peroxide	Increased	Enhanced the invasive and	[46,48]
		Increased	migratory activity	
Antioxidant	MnSOD	Decreased	Enhanced the invasive and migratory activity	[50,51]
enzymes	Catalase, glutathione peroxidase	Decreased		
		Decreased		
Cytokines and their receptors	GDNF and RET	Increased	Enhanced cell proliferation	[55]
	EGF	Increased	Enhanced cell proliferation	[56]
	EGFR	Transactivation	Enhanced cell proliferation	
	NGF	Increased	Aggravated the process of perineural invasion	[58]
	P75	Decreased		

*EGF*, epidermal growth factor; *EGFR*, epidermal growth factor receptor; *GDNF*, glial cell line-derived neurotrophic factor; *MnSOD*, manganese superoxide dismutase; *NGF*, nerve growth factor; *PanCa*, pancreatic cancer; *RAGE*, receptor for advanced glycation end products; *ROS*, reactive oxygen species; *uPA*, urokinase plasminogen activator.

In a microarray analysis of myoblasts cultured in PanCa cell-conditioned media, Basso *et al*. found that lactate production and induced proteolysis were enhanced in the myoblasts, which can induce hyperglycemia [44]. Hyperglycemia and oxidative stress can lead to the accumulation of advanced glycation end products (AGEs). Studies have reported the strong expression of RAGE in MiaPaCa-2 and Panc-1 that have high metastatic ability [45,46]. Reactive oxygen species (ROS) appear to be linked to PanCa. ROS have also been suggested to be mitogenic and capable of stimulating cell proliferation. In diabetic individuals, hyperglycemia in susceptible cells results in the overproduction of superoxide by the mitochondrial electron transport chain, and this process is the key to initiating all damaging pathways related to diabetes [47]. Hyperglycemia also specifically activates polyol metabolism with a consequent decrease in Na+, K + −ATPase activity in pancreatic duct epithelial cells [48]. Moreover, hyperglycemia enhances the invasive and migratory activity of pancreatic cancer cells via hydrogen peroxide and the increased expression of urokinase plasminogen activator (uPA) [49].

Hyperglycemia can attenuate antioxidant enzyme activity and in turn create a state of oxidative stress [50]. The PanCa lines BxPC-3, MiaPaCa-2, and AsPC-1 have decreased manganese superoxide dismutase (MnSOD) immunoreactive protein expression and activity, and decreases in MnSOD correlate well with increased rates of tumor cell proliferation as determined by cell doubling time [51]. Cullen *et al*. found that the cytoplasmic values of MnSOD, catalase, and glutathione peroxidase were decreased in pancreatic cells from chronic pancreatitis specimens when compared with normal pancreas [52]. Additionally, elevated fructose may directly contribute to oxidative stress in pancreatic cancer. Suzuki *et al*. showed that fructose increases H2O2 levels and lipid peroxidation of hamster islet tumor cells, which originated from hamster pancreatic beta cells. Furthermore, glutathione peroxidase (GPx) is inactivated by fructose, and the mRNA expression of GPx is suppressed by fructose [53].

Glial cell line-derived neurotrophic factor (GDNF) is a chemoattractant for pancreatic cancer cells in the processes of tumor progression, migration and invasion. In *in vitro* studies, Jemal *et al*. have confirmed the stimulating effect of GDNF on the proliferation and invasion of pancreatic cancer cells through the activation of the RET tyrosine kinase receptor [54,55]. Glucose alters the expression of GDNF and RET in a concentration-dependent manner, corresponding with the alterations in cell proliferation. Upregulation of the GDNF and RET ligand-receptor interaction might participate in the glucose-induced cancer progression [56]. High glucose also promotes PanCa cell proliferation via the induction of epidermal growth factor (EGF) expression and transactivation of EGFR [57].

The role of hyperglycemia in perineural invasion in pancreatic cancer is not clear. We have hypothesized that hyperglycemia promotes perineural invasion in PanCa by effects on nerve and cancer cells, respectively [58]. Our clinical study also showed that nerve damage and regeneration occur simultaneously in the tumor microenvironment of PanCa patients with hyperglycemia, thereby aggravating the process of perineural invasion. The abnormal expression of nerve growth factor (NGF) and p75 may also be involved in this process and subsequently lead to a lower rate of curative surgery [59].

### Diabetes and the early diagnosis of PanCa

Recently, there has emerged a growing understanding that new-onset diabetes type 2, which begins up to 24 months before cancer diagnosis, can be an early manifestation of asymptomatic PanCa [60]. The prevalence of diabetes is higher (40%) and more frequently of the new-onset type in PanCa than in control groups [61]. It has been suggested that screening for PanCa should be performed in patients with new-onset hyperglycemia and diabetes [62]. Because new-onset diabetes occurs 50 to 100 times more commonly than pancreatic cancer, screening efforts to detect occult pancreatic cancer in patients with new-onset diabetes require a feasible method to detect type 3 diabetes as the cause of impaired glucose tolerance [7]. Although the diabetes observed in patients with PanCa seems similar to type 2 diabetes in impaired glucose tolerance and insulin resistance, it affects only 1% to 5% of all diabetic patients [63]. Further support for the concept that the growing pancreatic tumor produces substances that alter glucose metabolism comes from experimental studies of glucose utilization and lactate formation in mouse myoblasts [44].

### Clinical manifestation of PanCa-associated diabetes

PanCa-associated diabetes has some specific characters in the clinic, which may be helpful for differentiation of PanCa-associated diabetes from type 2 diabetes (Table 4). Type 2 diabetes is common in the elderly and pancreatic cancer populations. In elderly subjects within two years of meeting criteria for diabetes, who have relatively low premorbid body mass index, experience weight loss, and have no family history of diabetes, a suspicion of pancreatic cancer is justified [64]. Impaired glucose tolerance or diabetes is often the first sign of PanCa and occurs shortly before other clinical symptoms occur, such as weight loss, malaise and pain [65]. Hart *et al*. compared body weight and fasting blood glucose at diabetes onset, one to two years before and at index date, and found that weight loss frequently precedes onset of PanCa-related diabetes, whereas new-onset primary type 2 diabetes is typically associated with weight gain, which may be an important clue to understanding the pathogenesis of PanCa-related diabetes [66]. Middle-aged diabetic men and women aged 45 to 65 years reportedly have the greatest risk of malignant neoplasms of the pancreas [67]. The prevalence of diabetes in PanCa did not differ by tumor stage or site. Median survival was similar in PanCa with and without diabetes [68]. Lin *et al*. found that PanCa should be considered in the list of precipitants for diabetic ketoacidosis in type 2 diabetes [69]. 

**Table 4:** The potential differentiation between PanCa-associated diabetes and type 2diabetes in the clinic

**Parameters**	**PanCa-** **related diabetes**	**Type 2 diabetes**	**Refs**
Age of onset	Aged 45–65 years	All ages	[66]
Body mass index	Relatively low	High	[63]
Family history of diabetes	No	Often	[63]
Body weight	Often loss	Often gain	[65]
Tumor stage and site	Similar	Similar	[67]

### Serological biomarkers

Biomarkers may be a useful strategy for the early diagnosis of PanCa, which can differentiate PanCa-associated diabetes from the more common type 2 diabetes and have differential expression the various stages in PanCa (Table 5).

**Table 5:** Biomarkers involved in PanCa cancer with diabetes

**Biomarkers**	**Site**	**Expression levels**	**Role**	**Refs**
IAPP	Serum	Increased	Early stage of PanCa	[28]
	Tissue	Decreased	Early stage of PanCa	[72,73]
Glucagon/insulin ratio	Serum	Increased	Identify PanCa in patients with new-onset DM	[74]
Adrenomedullin	Serum	Increased	Potential biomarker of PanCa-related DM	[75]
Fatty acid binding protein-1	Tissue	Increased	Diagnosis of PanCa	[76]
A 14-amino-acid peptide from S100A8	Serum	Increased	Identify PanCa in patients with new-onset DM	[38]
Vanin-1 and matrix metalloproteinase 9	Serum	Increased	Potential biomarker of PanCa-related DM	[77]
Higher CA 19–9 and/or CEA	Serum	Increased	Screen early PanCa	[79]
Erc/Mesothelin	Serum	Increased	Screen early PanCa	[81]

*CA*, cancer antigen; *CEA*, carcinoembryonic antigen; *DM*, diabetes mellitus; *IAPP*, islet amyloid polypeptide; *PanCa*, pancreatic cancer.

Perhaps the strongest evidence for the tumor itself as a source for diabetogenic factor(s) is derived from the observation that glucose metabolism is improved after resection of the PanCa [70]. The hypothesis that a tumor-associated factor elicits the development of diabetes in PanCa is also supported by the fact that the onset of diabetes predates the first symptoms of PanCa by as much as two years, that is, well before the tumor is large enough to induce degradation of pancreatic islets [71]. However, the use of a biomarker may help to differentiate, PanCa-associated diabetes from the more common type 2 diabetes [61]. IAPP causes hyperglycemia by inhibiting muscle glycogen synthesis and stimulating glucose release from the muscles [72]. Permert *et al*. found that plasma glucagon and IAPP were significantly increased at 12 and 27 weeks, respectively, demonstrating that islet hormone changes accompany the early development of pancreatic tumors in the hamster pancreatic model [29]. However, Saruc *et al*. compared the IAPP-expressing cells between cancer area and tumor-free area, and found a decrease rather than an increase in the number of IAPP-expressing cells in PanCa. The development of diabetes in subjects prone to pancreatic cancer could be a red flag for malignancy [73]. Katsumichi *et al*. also found that the number of IAPP-expressing cells was significantly lower in diabetes and in the tumor area but not in the tumor-free region [74].

Kolb *et al*. found significant differences in the serum glucagon/insulin ratio in patients with PanCa-associated diabetes and type 2 diabetes, with a borderline cut-off of 7.4 ng/mU glucagon/insulin [75]. Aggarwal *et al*. found that adrenomedullin (AM) is overexpressed in PanCa tissue and plasma, especially in association with diabetes. Additionally, plasma AM is elevated in PanCa. Further studies in a larger cohort of patients are planned to validate AM as a potential biomarker of PanCa-associated diabetes [76]. Using a novel translational bioinformatics approach, Sharaf *et al*. identified fatty acid binding protein-1 (FABP-1) as having a significant positive association with PanCa on tissue microarrays, which was further strengthened by the presence of diabetes [77]. Using microarray analysis and RT-PCR, Huang *et al*. demonstrated that the combination of vanin-1 and matrix metalloproteinase 9 may be used as a novel blood biomarker panel for the discrimination of pancreatic cancer-associated diabetes from type 2 diabetes [78]. In addition, Basso *et al*. isolated a 14-amino-acid peptide from S100A8 in PanCa tissue from PanCa patients with diabetes. As S100A8 impairs the catabolism of glucose by myoblasts *in vitro* and may cause hyperglycemia *in vivo*, it might be helpful in diagnosing PanCa in patients with recent-onset diabetes mellitus [39]. Selective stimulation of amylin secretion was demonstrated when isolated pancreatic islet cells were incubated in media conditioned with Panc-1 as compared to unconditioned media [79]. Cancer antigen (CA) 19–9 is used in the diagnosis of pancreatic cancer but is also a marker of pancreatic tissue damage that might be caused by diabetes. Thus, the diagnosis of new-onset diabetes combined with higher CA 19–9 and/or carcinoembryonic antigen (CEA) might be regarded as a useful tool to screen early pancreatic cancer [80].

Amadori-glycated phosphatidylethanolamine (Amadori-PE), known as a reliable indicator of lipid glycation *in vivo*, is a nonenzymatically glycated lipid formed under hyperglycemic conditions. Using the streptozotocin (STZ)-induced diabetic rat model, Sookwong *et al*. found high levels of Amadori-PE in the blood and in organs that are strongly affected by diabetes, such as the kidney with a significant increase in STZ rats seven days after STZ treatment, suggesting that Amadori-PE may be a useful predictive marker for early-stage diabetes [81]. Diabetes mellitus could be a risk factor for PanCa. Eitsuka *et al*. found that Amadori-PE-enhanced cellular telomerase, which contributed to the infinite replicative potential of Panc-1 cancer cells in a time- and dose-dependent manner by upregulating human telomerase reverse transcriptase (hTERT) expression through induction of c-myc. These results provide experimental evidence for a novel role of Amadori-PE in linking diabetes and PanCa [82].

Navaglia *et al*. introduced the technique of surface-enhanced laser desorption and ionization time-of-flight mass spectrometry, which permits identification of new peptides that, in addition to CA 19–9, enable the correct classification of the vast majority of patients with pancreatic cancer, who can be distinguished from patients with chronic pancreatitis or type 2 diabetes mellitus [83]. To identify biomarkers for early PanCa, Fukamachi *et al*. established transgenic rats carrying a mutated H-ras or K-ras gene as a PanCa model, and found that even in rats with very small microscopic ductal carcinoma lesions, elevated serum Erc/Mesothelin can be detected [84].

### Diabetes and the treatment for PanCa

Pelaez-Luna *et al*. determined the resectability of PanCa on abdominal computed tomography (CT) scans performed prior to clinical diagnosis and correlated resectability with onset of diabetes. They found that PanCa is frequently undetectable or resectable on CT scans performed ≥ 6 months prior to clinical diagnosis. At onset of diabetes, pancreatic cancers are generally resectable [85]. Several studies have reported the risk of antidiabetic drugs on cancer incidence. Chang *et al*. examined cancer incidence associated with use of insulin glargine, and found usage was positively associated with pancreatic and prostate cancers in men [86]. To elucidate the effect of pharmacotherapy for diabetes in cancer patients, Feng *et al*. found that insulin and glucose promoted cancer cell proliferation and contributed to chemoresistance, with metformin and rosiglitazone suppressing cancer cell growth and inducing apoptosis of four human cancer cell lines *in vitro*. Both drugs affected signaling in the protein kinase B (AKT)/mammalian target of rapamycin pathway, which provides experimental evidence to support further investigation of metformin and rosiglitazone as first-line therapies for type 2 diabetes in cancer patients [87]. Univariate and multivariate analyses were performed to determine whether the medications were predictive of early mortality in patients undergoing resection for PanCa.

Chagpar *et al*. found that patients with PanCa being treated for pre-existing diabetes with insulin therapy have an increased risk of early postoperative mortality [88]. To explore the ability of metformin to protect against cancer risk in Orientals, the possible metformin effect on total, esophageal, gastric, colorectal, hepatocellular and pancreatic cancers was examined in a Taiwanese cohort. In diabetics not taking anti-hyperglycemic medication, cancer incidence density increased at least twofold in total, including pancreatic cancers. The metformin dosage needed to observe a significant decrease in cancer incidence was ≤500 mg/day [89].

In addition to its ability to reduce insulin resistance, the antidiabetic drug, metformin has shown antitumor properties and is increasingly being considered as a drug for the prevention and treatment of obesity-related cancers [90]. Currie *et al*. reported that the effect of metformin might be tumor-specific, in that its use was associated with a reduced risk of cancer of the colon and pancreas, but not of cancer of the breast or prostate [91]. The antitumor effect of metformin seems to be mediated via its ability to increase the AMP-activated protein kinase (AMPK) signaling pathway [92]. Metformin negatively regulates mammalian target of rapamycin (mTOR) complex 1 (mTORC1) and the crosstalk between insulin/IGF-1 receptor and G-protein coupled receptor (GPCR) signaling, thus inhabiting the development of certain types of cancer [93].

Treatment with metformin induced striking and sustained increase in the phosphorylation of AMPK at Thr(172), and a selective AMPK inhibitor (compound C, at 5 μmol/L) reversed the effects of metformin on (Ca(2+))(i) and DNA synthesis, indicating that metformin acts through AMPK activation [94].

Using Cox proportional hazards models and controlling for smoking and alcohol use, a ten-year prospective cohort study reported that the stratum with the highest fasting serum glucose had higher death rates from all cancers combined compared with the stratum with the lowest level. By cancer site, the association was strongest for pancreatic cancer when comparing the highest and lowest strata in men and in women in Korea [95].

## Conclusions

Diabetes is widely considered to be associated with PanCa. Epidemiological studies have confirmed a higher incidence of diabetes in PanCa, while a higher incidence of PanCa in diabetes was also confirmed. These associations provide evidence-based medical support for further studies examining the mechanism of interaction.

Diabetes may directly promote the progression of PanCa by pancreatic duct enlargement and hypertension, as well as increased tumor size. Hyperinsulinemia and insulin resistance, cytokines, hyperglycemia and genotype change are also important factors involved in the mechanism of occurrence, proliferation, invasion, metastasis and prognosis of PanCa with diabetes. Up to 80% of PanCa patients are either hyperglycemic or diabetic, both of which can be detected in the presymptomatic phase. Hyperglycemia and glucose tolerance abnormalities may be the first clinical manifestation of diabetes, which can also be tested by a health examination. If the danger signal of hyperglycemia and glucose tolerance abnormalities can be emphasized, some of the hazard population of PanCa may be screened. Novel biomarkers of early PanCa are also being studied. The combination of defining a hazard population of PanCa and the use of novel biomarkers discriminating common type 2 diabetes from PanCa-associated diabetes should be the next direction in the early diagnosis of PanCa.

Antidiabetic drugs have different effects on the occurrence and prognosis of PanCa. Metformin can lower the risk of PanCa, the mechanism of which has also been explored, whereas insulin use exerts a converse effect. The novel usage of metformin for its antitumor effect and its potential mechanism may be another direction to improve the prognosis of PanCa.

## Abbreviations

AM: Adrenomedullin; Amadori-PE: Amadori-glycated phosphatidylethanolamine; AMPK: AMP-activated protein kinase; DM: Diabetes mellitus; EGF: Epidermal growth factor; EGFR: Epidermal growth factor receptor; GDNF: Glial cell line-derived neurotrophic factor; GPx: Glutathione peroxidase; hTERT: Human telomerase reverse transcriptase; IAPP: Islet amyloid polypeptide; IGF-1: Insulin-like growth factor 1; MnSOD: Manganese superoxide dismutase; mTOR(1): Mammalian target of rapamycin (complex 1); NGF: Nerve growth factor; PanCa: Pancreatic cancer; (R)AGE: (receptor for) advanced glycation end products; ROS: Reactive oxygen species; uPA: Urokinase plasminogen activator.

## Competing interest

The authors declare they have no competing of interest.

## Authors’ contributions

JHL, HL and WL wrote the manuscript, and GC, QYM and LH approved it. All authors read and approved the final manuscript.
